# How to prepare for a bright future of radiology in Europe

**DOI:** 10.1186/s13244-023-01525-3

**Published:** 2023-10-10

**Authors:** Minerva Becker

**Affiliations:** Unit of Head and Neck and Maxilofacial Radiology, Division of Radiology, Diagnostic Department, Geneva University Hospitals, University of Geneva, Rue Gabrielle Perret Gentil 4, Geneva 14, CH 1211 Switzerland

**Keywords:** Multidisciplinary collaboration, Patient-centred radiology, Strategic goals for radiology, Artificial intelligence in radiology, Radiology beyond reporting

## Abstract

Because artificial intelligence (AI)-powered algorithms allow automated image analysis in a growing number of diagnostic scenarios, some healthcare stakeholders have raised doubts about the future of the entire radiologic profession. Their view disregards not only the role of radiologists in the diagnostic service chain beyond reporting, but also the many multidisciplinary and patient-related consulting tasks for which radiologists are solicited. The time commitment for these non-reporting tasks is considerable but difficult to quantify and often impossible to fulfil considering the current mismatch between workload and workforce in many countries. Nonetheless, multidisciplinary, and patient-centred consulting activities could move up on radiologists’ agendas as soon as AI-based tools can save time in daily routine. Although there are many reasons why AI will assist and not replace radiologists as imaging experts in the future, it is important to position the next generation of European radiologists in view of this expected trend. To ensure radiologists’ personal professional recognition and fulfilment in multidisciplinary environments, the focus of training should go beyond diagnostic reporting, concentrating on clinical backgrounds, specific communication skills with referrers and patients, and integration of imaging findings with those of other disciplines. Close collaboration between the European Society of Radiology (ESR) and European national radiologic societies can help to achieve these goals. Although each adequate treatment begins with a correct diagnosis, many health politicians see radiologic procedures mainly as a cost factor. Radiologic research should, therefore, increasingly investigate the imaging impact on treatment and outcome rather than focusing mainly on technical improvements and diagnostic accuracy alone.

**Critical relevance statement **Strategies are presented to prepare for a successful future of the radiologic profession in Europe, if AI-powered tools can alleviate the current reporting overload: engaging in multidisciplinary activities (clinical and integrative diagnostics), enhancing the value and recognition of radiologists’ role through clinical expertise, focusing radiological research on the impact on diagnosis and outcome, and promoting patient-centred radiology by enhancing communication skills.

**Key points • **AI-powered tools will not replace radiologists but hold promise to reduce the current reporting burden, enabling them to reinvest liberated time in multidisciplinary clinical and patient-related tasks.

• The skills and resources for these tasks should be considered when recruiting and teaching the next generation of radiologists, when organising departments and planning staffing.

• Communication skills will play an increasing role in both multidisciplinary activities and patient-centred radiology.

• The value and importance of a correct and integrative diagnosis and the cost of an incorrect imaging diagnosis should be emphasised when discussing with non-medical stakeholders in healthcare.

• The radiologic community in Europe should start now to prepare for a bright future of the profession for the benefit of patients and medical colleagues alike.

**Graphical Abstract:**

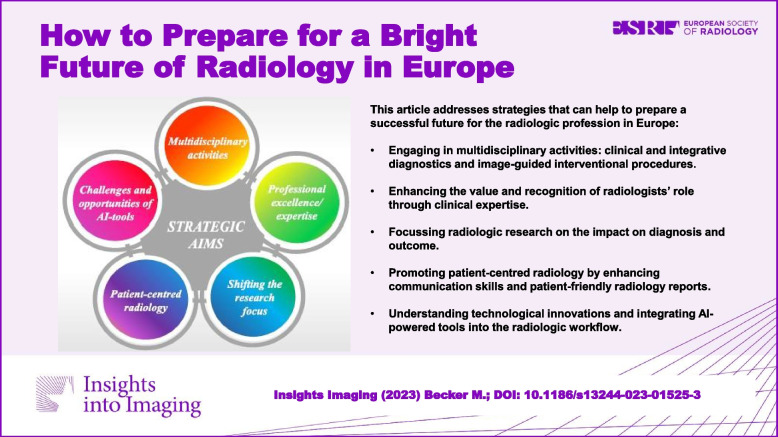

## Introduction

After several decades of unprecedented growth and development of radiology at the forefront of digitisation in medicine, radiologists have become victims of the success of their own discipline, resulting in an imbalance between excess workload and lack of available workforce in many European countries [1–3]. At the same time, radiology is commonly cited among the data-driven professions which are thought to be soon and profoundly impacted by the developments of artificial intelligence (AI) and, more specifically, deep learning (DL). Predictions by some stakeholders already foretell that AI-powered algorithms will provide analysis of most medical images in the future, as well as automated reports directly to the referring physician, thus rendering the radiologist’s role unnecessary [4, 5]. Unsurprisingly, the paradoxical image of the modern radiologist being caught between the risks of burnout today and obsolescence tomorrow leads to doubts and uncertainties about the future of the entire specialty. Together with political trends towards austerity in healthcare already observed in some European countries, such prospects may become discouraging for young colleagues who consider a radiologic career thus leading to a self-fulfilling gloomy prophecy. Also, those who are responsible for providing radiologic services to patients and for structuring resident programmes and staff careers for the next decades face the difficult task of planning without knowing the pace and the extent of integration of AI-powered tools into the practical clinical workflow. In addition, the current shortage of adequately trained and subspecialised radiologists has also increased the popularity of teleradiological outsourcing in some European countries, thus contributing to diagnostic radiology being perceived as a commodity and the radiologist as being “invisible” [6–9].

Much of this pessimism about the radiologic profession is based on viewing the radiologists’ role as being limited to reading and reporting diagnostic studies. However, this reduced view disregards much of the radiologist’s activities that add value to the diagnostic service chain, namely, optimising quality and safety though a patient-centred approach, providing multidisciplinary and consulting services for clinical colleagues, and participating in modern treatments through image-guided minimally invasive interventions [6, 10–13]. As soon as AI-powered tools will be ready to be integrated on a large clinical scale and become helpful to alleviate some of the current overwhelming reporting burden, radiologists may be able to move their role as imaging consultants up on their agenda, allocating the necessary time to the non-reporting tasks for which they are already solicited today.

**Fig. 1:**
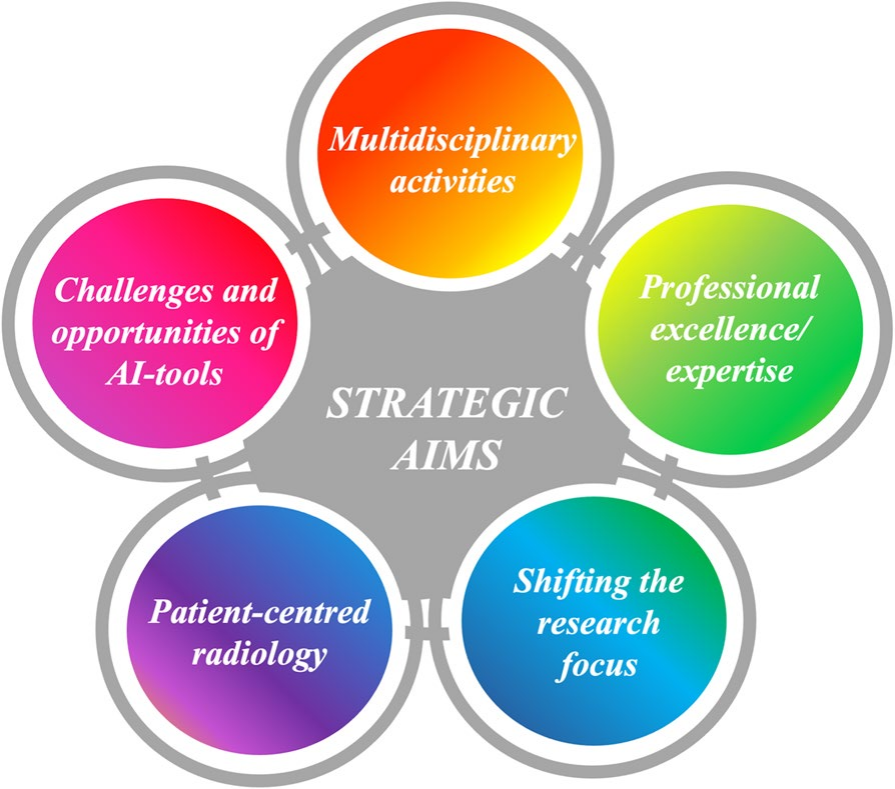
Synopsis of strategies aiming for a bright future in the radiologic profession

Some strategies that may help to prepare a successful future for the radiologic profession in Europe are addressed in this article (Fig. 1).

## Engaging in multidisciplinary activities: clinical and integrative diagnostics and image-guided interventional procedures

Collaboration between different medical disciplines is indispensable in modern medicine and plays a key role in optimal clinical results. Because of the transverse character of their discipline, radiologists interact with many medical specialties and subspecialties and in many formats, including regular clinical rounds, multidisciplinary meetings and tumour boards, second opinion services, assessment of longitudinal follow-up, or planning of minimally invasive treatments [11, 14–16]. The importance that European radiologists assign to their multidisciplinary consulting activities has been shown in recent surveys by the European Society of Radiology (ESR) and the European Society of Oncologic Imaging (ESOI) [14, 17]. In many European countries, participation of experienced and subspecialised radiologists in decisional multidisciplinary tumour boards is required for the accreditation of specialised centres, especially in oncology [17, 18]. Furthermore, many radiologists are also commonly solicited ad hoc by their colleagues to answer specific questions about complex imaging findings and for second opinions about examinations from outside institutions.

Multidisciplinary collaboration requires the radiologist’s ability to “speak the same language” as their clinical colleagues and to be aware of the clinical implications of image-based diagnosis. Therefore, it is important for radiologists in training to participate regularly in different clinical decisional conferences. They should learn to provide actionable, clinically relevant information on which treatment decisions can be based, ideally in a standardised or a structured report format rather than making lengthy descriptions of technical parameters such as attenuation values, signal behaviour, or echogenicity of structures [18, 19]. Radiologists who work in settings in which multidisciplinary collaboration is a major part of their activity will usually develop a profile that mirrors the degree of specialisation of their clinical referrers, including not only specific knowledge of the clinical background but also familiarity with the respective international multidisciplinary disease classification and quantification systems. With these competences, they will not only provide significant clinical value, but they will also be more likely to earn the respect of their referrers than through mere technical expertise or descriptive reporting.

Although the clinical value and the necessary expertise for all the above-mentioned activities have been well documented, the associated time commitment remains difficult to measure and is, therefore, often unsatisfactorily reflected in current staffing and reimbursement models [11, 13–16]. The ESR and the European national radiologic societies should join efforts to convince policymakers and hospital administrators of the importance of radiologic expertise. It is important to understand that a correct diagnosis is not only the basis for correct treatment, but that each incorrect diagnosis leading to incorrect treatment is a source of hidden costs both for patients and society [20–25]. Since the dialogue between radiologists and referrers and in the context of multidisciplinary activities may enhance the quality of radiologic services to patients, it is very important to understand the drawbacks of the commoditisation of radiologic services through teleradiological outsourcing. Although being welcomed by some hospital administrators and health politicians for apparent economic reasons, the commoditisation of diagnostic imaging tends to hamper the interdisciplinary dialogue, and the unavailability of teleradiologists at multidisciplinary meetings precludes the development of a relationship of trust with their clinical colleagues. This may either compromise the value of diagnosis or require additional workload for on-site radiologists, thus creating another source of hidden cost [26, 27].

Another aspect of multidisciplinary collaboration is the convergence towards integrated diagnostics, a term referring to the concept of comparing complex diagnostic results in detail, detecting discrepancies, and creating consensus among different diagnostic specialists with the goal of providing a common final diagnosis. The collaboration between radiologists and nuclear medicine physicians has been greatly facilitated by hybrid imaging modalities which combine positron-emission tomography (PET) with computed tomography (CT) or magnetic resonance imaging (MRI). Correlation of radiologic and pathological findings is a well-established and fascinating method for didactic and research purposes [28–31]. However, it is not yet well established in clinical routine, because radiologists and pathologists often work in different professional environments or “silos” with different tools and different workflows. Progress in IT platforms and infrastructure and the trend towards digitisation of pathology may help to facilitate this collaboration [32, 33]. Some major European teaching hospitals have taken the first steps towards this organisational form by regrouping nuclear medicine, pathology, and clinical genetics or laboratory medicine together with radiology in common diagnostic departments. However, considerable technical and managerial efforts are still necessary to make this vision become reality. Finally, successful implementation of integrated diagnostics requires the willingness of different specialists to go beyond traditional schemes and leave their traditional professional silos in order to collaborate [32–34]. Notwithstanding these obstacles, it appears worthwhile to pursue the goal of integrated diagnostics because it can improve not only diagnostic precision but also the efficiency of multidisciplinary tumour boards during which too much time is often devoted to bringing diagnostic facts together rather than focusing on treatment decisions.

Interventional radiologic procedures are now well established as minimally invasive alternatives to surgical or endoscopic techniques in many diagnostic and therapeutic settings. Interventional radiologists participate actively in patient care, and since many modern interventional procedures are complementary to or combined with other forms of treatment, they are naturally integrated into multidisciplinary collaborations. Rather than simply executing procedures upon request, interventional radiologists should always participate in clinical decisions, consider the benefits and risks of their procedures, and adequately manage any complications together with their clinical colleagues [35].

For all the above-mentioned reasons, it is essential for the radiologic community throughout Europe to enhance their interest, value, and commitment for multidisciplinary collaboration and to prepare future radiologists for their role as imaging consultants in the different organ- and pathology-based domains, especially in oncology and in other areas of personalised precision medicine [36, 37].

## Enhancing the value and the recognition of the radiologist’s role through clinical expertise

Excellence in professional performance though adequate training is an indispensable prerequisite and the key factor for successfully positioning radiology as an essential medical discipline for the future. The current ESR training curriculum [38] is quite complete and regularly updated and may serve as a guideline throughout Europe. Future revisions should address questions as to how and when competence-based objectives (“skills”) should be reached and tested in residency programmes by defining, developing, and implementing so-called entrustable professional activities (“EPAs”) for both diagnostic and interventional radiologic procedures. However, radiology training programmes in Europe are most often defined by national bodies and, therefore, subject to general political trends. EPAs have been introduced or are planned for the next years in about one-half of European countries [39]. Recently, a tendency is observed in some countries to shorten the dedicated radiology training periods for apparent economic reasons. However, the reduction of training periods for national radiology boards may have negative effects on the overall professional performance of future radiologists. It, therefore, appears important for the ESR and the national radiologic societies to make a joint effort to explain to national regulatory bodies in Europe, where necessary, that the learning objectives for the European Diploma of Radiology (EDiR) (levels I–II) are to be met for quality reasons, that acquisition of knowledge and skills required for the radiology board usually takes no less than 5 years of training, and that each erroneous diagnosis may lead to hidden costs through inadequate treatment [20, 40].

Excellence in radiologic professional performance is also essential for turf issues about medical imaging, although these will mainly be solved or decided on a local or national basis rather than on a European level. It appears elusive and unrealistic for radiologists to aim at a monopoly for all imaging techniques. This applies also to situations in which referring physicians prefer to perform image-guided interventional procedures themselves rather than requesting radiologists to do so. In many European countries, non-radiologic clinical specialists routinely use ultrasonography, interpret standard radiography, or perform procedures under imaging guidance, and some specialties even claim to “co-own” computed tomography (CT) and magnetic resonance imaging (MRI) by including these imaging modalities into their own postgraduate training curricula and offering individual accreditation [41, 42]. Even when “owning” the imaging infrastructure for CT and MRI or interventional angiography, the only way for radiologists to remain competitive in many turf issues is through subspecialisation according to the mainly organ-based clinical disciplines. Therefore, implementation of radiologic subspecialisation in Europe according to the level III curricula (beyond year 5) should not be limited to large academic departments but is necessary wherever subspecialised medical services are being regularly provided. A recent survey among ESR members has emphasised that the acquisition of a radiologic subspecialty accreditation was considered to play a key role to gain recognition from clinical colleagues, and to enhance visibility and professional fulfilment as a member of a multidisciplinary organ-based clinical service [17]. However, the concept of subspecialised radiology necessitates the creation of senior staff positions, as well as the necessary organisation and administrative workforce to cover each organ-specific service throughout the year. This does not mean that a full set of radiologic subspecialists is required in every regional or community hospital. But it does mean that it becomes more and more important for any radiologic service to match the specialty profile of the referring clinicians in a given local setting [43]. Although this has already been accomplished widely and beyond teaching hospitals in North America, it has only been incompletely adopted in Europe today. Implementation of this concept throughout Europe could be supported by a joint effort by both the ESR and the European radiologic national societies. This could be done by propagating the recognition of the different ESR-endorsed subspecialty board diplomas, including the European Board of Interventional Radiology (EBIR) by national organisms.

## Focusing radiologic research on the impact of imaging diagnosis and outcome

In the current European healthcare environment, which is characterised by political efforts towards cost containment, diagnostic procedures are often cited as a cost factor. It is, therefore, important to provide scientific evidence to define the clinical role of diagnostic imaging in specific pathologies and disease processes. Although a major part of today’s radiologic research focuses on technical aspects and accuracy of imaging procedures, future studies should focus more often on questions related to the impact of imaging on diagnosis and treatment outcome [44–46]. Outcome-related research will not only serve as a useful basis for the development of clinical decision tools for imaging prescription such as the ESR i-guide [47] in order to avoid unnecessary prescriptions but may also help to recognise the value of imaging to avoid hidden costs associated with an incorrect diagnosis and to enhance personalised therapeutic approaches. The ESR could support this goal strategically by allocating and directing research funds specifically to outcome-related research and by enhancing the visibility of such research through publications in the ESR journals. As pointed out in a recent editorial in this journal, critical thinking, i.e. the ability to analyse, question, interpret, and test established facts and information, is an essential step towards strengthening radiologic research [48].

## Promoting patient-centred radiology by enhancing communication skills

Direct contact between radiologists and patients occurs naturally and routinely, not only before and after interventional procedures, but also in many settings of diagnostic imaging, e.g. ultrasonography, mammography, or fluoroscopy. Direct communication between radiologists and patients also reduces the risk of relevant information remaining unspecified or even lost at the time of prescription, thus limiting the quality of diagnosis [49, 50]. According to the traditional flow of information, imaging results are usually communicated to patients by the referring clinicians rather than by the radiologist who has made the diagnosis. Following the trends of modern healthcare, however, future generations of patients will increasingly access their personal health data including radiology reports and images directly through digital patient portals [51–54]. Being better informed about their personal health data, they may also wish to participate more actively in therapeutic decisions. Although many referring clinicians are able to answer simple questions or interpret conventional radiographs in their domains, they may not be able to answer patients’ detailed questions about advanced imaging studies and complex findings [46]. Radiologists should, therefore, be prepared to respond to patients’ questions when solicited to do so. The necessary competences and attitudes include soft skills, such as the ability to relate to patients and their families with empathy, respect, honesty, and confidentiality in the presence of either good or bad diagnostic news, while leaving therapeutic aspects to the referring clinician. Although skills regarding direct communication with patients are mentioned among the learning objectives of the European training curriculum, many radiologists in Europe still feel that they lack the necessary communication skills, indicating that improvement through specific training courses is needed [55]. A special situation exists regarding interventional radiology, where patients may expect to consult with a doctor, who cannot only deliver a procedure, but who is also closely involved in pre-procedural assessment, the informed consent process, and follow-up both after the procedure and subsequently post-discharge [35].

Direct access of patients to their health data also creates a new audience for radiology reports. Referring physicians require well-structured, actionable reports in precise scientific quantitative terms, including recommendations for further diagnostic workup, where appropriate [19]. However, patients consulting their medical files may prefer consumer health language and hyperlinks explaining anatomical or pathological terms. Together with the IT industry and including patient organisations, the radiologic community should help to pave the way towards automated generation of radiology reports to enable patients who consult their radiologic files online to understand the impact of imaging diagnosis on their treatment processes. Finally, today’s radiology departments often lack a dedicated infrastructure allowing for direct communication between radiologists and patients. In the future, radiology departments should, therefore, provide appropriate solutions in their organisation and infrastructure to allow patients to get in contact with imaging specialists for obtaining explanations either in written form or by online or face-to-face meetings, whenever appropriate [54, 55].

## Understanding technological innovations and integrating AI-powered tools into the radiologic workflow

In their role as imaging specialists, radiologists must be able to understand basic technical principles related to medical imaging in many different areas of biomedical engineering and data science. This is necessary to critically appraise and integrate emerging technologies, to communicate with vendors and to participate in guiding further innovations [56]. Since the development of medical imaging is driven to a major part by digital data science, there is no doubt that AI will impact the practice of radiology profoundly in the future. Several industrial vendors have already integrated AI for MR image acquisition, reducing acquisition times without compromising quality. The software industry already offers many AI-powered tools to optimise workflows, facilitate image postprocessing and quantitative analysis, and automatically detect a variety of diagnostic findings in a large spectrum of use case scenarios in clinical radiology including common emergency situations such as brain haemorrhage, pulmonary embolism, or pneumothorax, to identify radiographs without pathologic findings or for screening purposes [2, 5, 57]. In view of their current overwhelming reporting workload, radiologists should, therefore, embrace the assistance of AI-powered tools as far as these can reduce the burden related to routine image interpretation. Although some published results indicate that the workload may be reduced in certain clinical scenarios, e.g. mammography sceening, there is currently little scientific evidence regarding the ability of these self-learning diagnostic tools to reduce the radiologists’ workload on a wider or general scale [58–60].

Apart from simple questions and typical findings, radiologic image interpretation is an opinion-based and operator-dependent process which is guided by a clinical context. In contrast, AI-based algorithms are based on mathematical models and probabilities, and the exact process of decision-making remains unknown, resembling a black box. To become reliable, algorithms using DL must be trained with very large volumes of data, and scientific evidence must be established with adequate reference standards. It can, therefore, be expected that DL-based tools will become useful primarily for the detection of common and clearly defined standardised diagnostic findings with a high volume in clinical practice. It appears very unlikely, however, that algorithms and robots can—in a foreseeable future—solve the entire range of complex, uncommon, and borderline diagnostic situations, with which radiologists are confronted, nor that they can take over the role as an imaging consultant in the vast field of diagnostic imaging [46]. It appears rather likely that the two different approaches will be used in a complementary fashion, thus combining their respective advantages. Furthermore, important questions still remain to be answered regarding the general use of AI-powered tools in radiology, e.g. the liability in case of diagnostic errors, the scientific evidence levels of performance in the context of self-learning devices, and many regulatory aspects including data protection throughout Europe [61]. Only time can tell when all these remaining obstacles will be overcome before the full potential of DL-based diagnostic algorithms will be deployed in clinical routine [62]. In the meantime, the radiologic community is well advised to understand and critically appraise the advantages and shortcomings of AI-based tools. Therefore, basic knowledge about this new technology has already been integrated into the ESR training curriculum. Finally, the ESR may play an important role in the related discussions with all European stakeholders including industry, by promoting future research into defining where and how AI-powered tools can save time without compromising quality in clinical practice.

## Conclusion

It appears very unlikely that algorithms and robots will replace radiologists in clinical practice, but there is hope that in the foreseeable future, AI-powered tools will be able to help radiologists cope with their currently overwhelming reporting burden. Once accomplished, this should progressively allow radiologists to liberate the necessary time—which is currently lacking—for the multidisciplinary and patient-related consulting tasks for which they are solicited, thus becoming more actively involved in patient care. This should be taken into consideration when recruiting and teaching the next generation of radiologists, when organising future radiology departments, when defining collaborations between the ESR and national radiologic societies, when discussing professional issues about radiology with hospital administrators and health politicians, and when prioritising research for funding. The time to address the above-described agenda is now. By accomplishing these strategic tasks, the European radiologic community can prepare for a bright future in the profession for the benefit of patients and medical colleagues alike!

## Data Availability

Not applicable.

## Abbreviations

AI: Artificial intelligence
CT: Computed tomography
DL: Deep learning
EBR: European Board of Radiology
EDiR: European Diploma in Radiology
EPA: Entrustable Professional Activities
ESOI: European Society of Oncologic Imaging
ESR: European Society of Radiology
IT: Information technology
MRI: Magnetic resonance imaging
PET: Positron emission tomography
